# Clinical Utility of Circulating Tumour DNA (ctDNA) Analysis for Assessing Completeness of Primary Lesion Resection and Disease Stage in Patients with Melanoma: A Systematic Review

**DOI:** 10.3390/medicina62030461

**Published:** 2026-02-28

**Authors:** Monika Wojarska, Klaudia Kokot, Paulina Bernecka, Aleksandra Kierczak, Natalia Sitkiewicz, Aleksandra Wakszyńska, Tomasz Wichowski, Weronika Skok, Milena Matwiejczuk, Wiktor Lijewski, Jerzy Jankau

**Affiliations:** 1 Plastic Surgery Department, University Clinical Center in Gdańsk, 80-952 Gdańsk, Poland; jerzy.jankau@gumed.edu.pl; 2 Students’ Scientific Circle of Plastic Surgery, Plastic Surgery Department, Medical University of Gdańsk, 80-210 Gdańsk, Poland; pauline.bernecka@gmail.com (P.B.); olakierczak@gumed.edu.pl (A.K.); natalia.sitkiewicz@gumed.edu.pl (N.S.); wakszynska@gumed.edu.pl (A.W.); tomasz.wichowski@gumed.edu.pl (T.W.); skok.wera@gumed.edu.pl (W.S.); milena.matwiejczuk@gumed.edu.pl (M.M.); wiktor.lijewski@gumed.edu.pl (W.L.); 3 Scientific Circle of Neurotraumatology, Department of Emergency Medicine, Medical University of Gdańsk, 80-210 Gdańsk, Poland

**Keywords:** melanoma, circulating tumour DNA, ctDNA, cell-free DNA, cfDNA

## Abstract

*Background and Objectives:* Melanoma is an aggressive cutaneous malignancy with a high recurrence rate even after complete resection. Circulating tumour DNA (ctDNA) has emerged as a promising biomarker for detecting minimal residual disease (MRD), assessing tumour burden, and predicting recurrence. This study aims to evaluate the clinical utility of ctDNA analysis in determining completeness of melanoma resection and disease staging. *Materials and Methods:* A systematic review was conducted in accordance with PRISMA guidelines, searching PubMed and Web of Science for studies published between January 2017 and February 2025. Eligible studies assessed ctDNA before, during, or after melanoma resection to evaluate surgical completeness and staging. Studies without perioperative ctDNA assessment or which focused solely on immunotherapy efficacy were excluded. *Results:* Fourteen studies with 1077 patients met the inclusion criteria. Preoperative ctDNA detection correlated with advanced stage, greater tumour burden, and poorer survival. Postoperative ctDNA persistence was strongly associated with recurrence, often detectable months before clinical relapse. In most patients remaining disease-free, ctDNA cleared within weeks after surgery. ctDNA levels reflected metastatic spread, though sensitivity was lower for brain lesions. Across studies, undetectable postoperative ctDNA was consistently linked to longer recurrence-free survival. *Conclusions:* Perioperative ctDNA analysis shows promise as a prognostic biomarker for detecting residual disease and anticipating relapse in melanoma. However, heterogeneity in patient cohorts, study design, and ctDNA detection methods limits immediate clinical application. Large, standardized prospective trials are needed to validate ctDNA for perioperative management.

## 1. Introduction

### 1.1. Melanoma

Melanoma is the most aggressive form of skin cancer associated with the highest mortality rate among cutaneous malignancies [1,2,3,4]. It is a malignant tumour arising from melanocytes—pigment-producing cells located mainly in the basal layer of the epidermis, but also, less frequently, in mucous membranes and the cornea. More than 90% of melanomas are cutaneous [5,6,7].

Several experimental and translational studies have demonstrated that melanogenesis profoundly influences melanoma biology through oxidative, metabolic, and immunological mechanisms. Active melanogenesis has been implicated as an important biological process contributing to malignant transformation of melanocytes and melanoma progression. Enhanced melanogenic activity results in the generation of free radicals and highly reactive intermediates with documented genotoxic and mutagenic properties [8,9,10,11,12,13]. Moreover, melanin pigments and their monomers—pheomelanin in particular—may, under specific conditions, promote a pro-oxidative environment and induce DNA damage [14,15,16,17,18]. In addition, melanin has been shown to exert direct pro-inflammatory and pro-oxidant effects on keratinocytes, independent of ultraviolet radiation exposure [19]. Consequently, dysregulated melanogenesis occurring within melanosomes or via the auto-oxidation of soluble intermediates may deplete intracellular antioxidant reserves and generate reactive oxygen species, while quinone and semiquinone intermediates can directly interact with RNA, DNA, and regulatory proteins, collectively creating a pro-mutagenic microenvironment conducive to melanomagenesis [20].

The global incidence of melanoma continues to rise, with approximately 25 new cases per 100,000 inhabitants in Europe, 30 per 100,000 in the United States, and up to 60 per 100,000 in Australia and New Zealand [21,22]. In Poland, about 3800 new melanoma cases are diagnosed annually, of which nearly 1500 present at an advanced or disseminated stage [23]. By 2040, melanoma is projected to become the most common cancer in men in the United States and the second most common in the general population [24]. Approximately 75% of cases are directly attributed to increased exposure to mutagenic ultraviolet (UV) radiation, which induces oncogenic alterations leading to malignant transformation [25,26,27].

Several molecular signalling pathways play a crucial role in melanoma initiation and progression. Mutations in BRAF and NRAS are critical components of the mitogen-activated protein kinase (MAPK) pathway. Among these, BRAF mutations represent the most important therapeutic target and occur in 40–60% of cutaneous melanomas [28,29,30,31].

Melanoma progression is also linked to metabolic reprogramming. As with many malignant tumours, melanoma cells rely heavily on aerobic glycolysis to support growth and progression [32,33,34,35], and alterations in glucose metabolism are central to tumour evolution and therapeutic resistance [33,36,37]. Melanin synthesis consumes molecular oxygen [38,39], while the melanogenic intermediate L-DOPA has been shown to stimulate glycolysis in melanotic melanomas [40] and activate the pentose phosphate pathway [41]. Furthermore, melanogenesis and L-DOPA oxidation have been associated with substantial changes in glycoprotein phosphorylation patterns [42]. Metabolomic analyses using high-resolution magic angle spinning nuclear magnetic resonance have demonstrated that induction of melanogenesis is accompanied by alterations in glucose and acetate metabolism [43]. Experimental studies have further revealed that melanogenesis induces accumulation of hypoxia-inducible factor-1α (HIF-1α) and robust upregulation of both HIF-1-dependent and independent signalling pathways, suggesting a direct role for melanogenesis in regulating melanoma cell metabolism and biological behaviour [44]. Supporting these findings, immunohistochemical analyses have demonstrated higher expression of HIF-1α and GLUT-1 in advanced melanomas compared with melanocytic nevi or thin cutaneous melanomas.

In addition to metabolic effects, melanin precursors not only stimulate melanogenesis itself [45,46,47] but also enhance the expression and activity of its regulatory pathways, including melanocyte-stimulating hormone receptors [45,48,49], as well as the production of proopiomelanocortin (POMC) and POMC-derived peptides [50]. Importantly, POMC-derived peptides, including the melanocyte-stimulating hormone, possess immunosuppressive properties [51,52,53], and increased expression of these peptides has been observed during melanoma progression to advanced stages [54,55,56,57,58].

Collectively, the stimulation of melanogenesis promotes a pro-oxidative and mutagenic cellular environment and induces metabolic rewiring characterized by enhanced glycolysis and HIF-1α activation. When combined with the immunosuppressive effects of melanogenic signalling, these processes may facilitate melanoma progression and contribute to resistance to immunotherapy, chemotherapy, and radiotherapy. The biophysical properties of melanin itself may further attenuate therapeutic efficacy. Accordingly, the inhibition of melanogenesis in advanced melanotic melanomas has been proposed as a potential strategy to sensitize tumours to existing treatment modalities or to directly limit tumour growth [20].

Notably, the role of melanin pigmentation in melanoma progression remains complex and context-dependent. Experimental data suggest that melanosomes may influence the migratory capacity of melanoma cells in vitro [59], and follow-up studies have shown that melanin-containing melanoma cells exhibit reduced metastatic potential in nude mouse models compared with non-pigmented cells [60]. These observations raise the possibility that melanin may inhibit certain steps of the metastatic cascade, although it remains unclear whether in vivo stimulation or inhibition of melanogenesis would ultimately promote or suppress metastasis. Under in vitro conditions, melanogenesis has been shown to alter adhesive properties of melanoma cells and promote detachment of heavily pigmented cells from the substratum [44,61,62,63], a process that could theoretically facilitate tumour cell dissemination in vivo. Therefore, further studies are required to delineate the relative contributions of melanogenesis-induced oxidative stress, the biochemical activity of melanin, and the mechanical properties of melanin granules in regulating melanoma progression and metastatic spread [20].

Diagnosis of melanoma can be initiated with dermatoscopy but requires histopathological confirmation, complemented by immunohistochemical and molecular analyses, which together constitute the diagnostic gold standard [6,64]. The most effective approaches to melanoma management include prevention, routine screening, early diagnosis, and surgical excision when the disease is limited to the skin [25,65,66,67,68]. The primary treatment for cutaneous melanoma is complete surgical excision with histologically confirmed clear margins. The recommended margins are 1 cm for tumours ≤ 1.0 mm in thickness (T1) and 2 cm for tumours > 2 mm (T3–T4) [23].

Management also includes the evaluation of regional lymph nodes. Sentinel lymph node biopsy is the standard approach for detecting subclinical nodal metastases [23,69]. In cases of proven nodal involvement without distant metastases, lymph node dissection is performed concurrently with resection of the primary tumour [70]. All patients with invasive melanoma should undergo systemic staging to identify additional primaries, satellite or in-transit metastases, and nodal involvement [23].

Imaging methods such as ultrasound, magnetic resonance imaging (MRI), and positron emission tomography–computed tomography (PET-CT) are currently recommended for staging and treatment monitoring in patients with stage IIB melanoma or higher [71]. Postoperative recurrences are common, especially in patients with resectable high-risk melanoma (stages IIB–IIC, IIIB–IIID, IV) [72].

Systemic treatment in the perioperative setting may be delivered either as adjuvant therapy, administered following surgery, or as neoadjuvant therapy, given prior to surgical excision.

Adjuvant therapy with anti-PD-1 antibodies or BRAF-targeted agents is now the standard of care in resectable high-risk melanoma, significantly reducing the risk of recurrence and improving distant metastasis-free survival [73]. Neoadjuvant immunotherapy is an emerging approach for stage III resectable melanoma, though it is not yet implemented in routine practice [74].

Despite therapeutic advances, recurrence rates remain high, underscoring the need for novel prognostic and predictive tools. Among the emerging biomarkers, circulating tumour DNA (ctDNA) has recently emerged as a promising tool for identifying minimal residual disease (MRD), monitoring tumour burden, and predicting recurrence, potentially enabling earlier therapeutic intervention.

### 1.2. ctDNA

Research on circulating DNA from solid tumours has provided new perspectives in oncology. Circulating cell-free DNA (cfDNA) consists of short DNA fragments detectable in body fluids such as plasma [75]. In cancer patients, a portion of cfDNA is derived from tumour cells originating from the primary lesion, metastases, or circulating tumour cells [76].

The release of ctDNA is linked to cellular damage or apoptosis, although the exact mechanism remains incompletely understood. ctDNA has a short half-life ranging from 16 to 139 min [77]. Its prognostic value has been demonstrated in various malignancies, including breast, lung, colorectal, oesophageal, bladder, and pancreatic cancers [77].

The analysis of ctDNA, commonly referred to as a “liquid biopsy,” represents a promising alternative to traditional tissue biopsy. This approach allows for the detection of minimal residual disease (MRD), which may drive future recurrence. As in other solid tumours, melanoma cells release ctDNA into the bloodstream and other body fluids, and molecular characterization of these fragments can provide valuable diagnostic and therapeutic insights.

Methods for ctDNA analysis vary in scale, ranging from the detection of single mutations to comprehensive genomic profiling. ctDNA detection relies on two primary methodological approaches: PCR-based techniques and next-generation sequencing NGS-based technologies. Droplet digital PCR (ddPCR) enables highly sensitive and quantitative detection of ctDNA by partitioning DNA samples into thousands of droplets and amplifying target sequences individually [78]. This approach allows precise detection of known mutations at very low allele frequencies, with reported detection limits of approximately 0.1% ddPCR [78]. This method is relatively rapid, cost-effective, and well suited for routine diagnostic applications. However, its high specificity restricts its clinical utility to predefined, known mutations, limiting its ability to identify novel or unexpected genomic alterations. In contrast, NGS-based approaches provide broader genomic coverage and enable simultaneous detection of multiple mutations. NGS can be performed using targeted gene panels, such as amplicon-based sequencing, which focuses on predefined genomic regions of clinical relevance and offers a favourable balance between sensitivity, cost, and turnaround time. While amplicon-based NGS is highly sensitive and suitable for clinical workflows, mutation detection remains limited to the targeted regions [79]. Consequently, the choice of ctDNA detection method can substantially influence detection rates and reported sensitivities across studies. This methodological heterogeneity represents a key source of variability and provides a rationale for why quantitative pooling of results across studies using different ctDNA detection techniques may not be feasible.

The application of ctDNA in melanoma includes its potential use in early detection. Early diagnosis significantly improves survival [80]. However, in Poland, nearly half of new melanoma cases are identified at advanced stages [81]. Challenges to implementing ctDNA-based early detection include tumour heterogeneity, growth dynamics, metastatic timing, and the cost-effectiveness of routine liquid biopsy [75,82].

In advanced melanoma, ctDNA analysis can help characterize tumour heterogeneity and provide prognostic information.

A significant proportion of melanoma patients treated surgically eventually experience recurrence. The likelihood of local relapse or distant metastasis is strongly correlated with tumour size [83]. Even after radical excision and adjuvant therapy, disease recurrence often occurs due to the persistence of MRD, which serves as a reservoir for metastatic spread [84].

Perioperative ctDNA analysis in patients with resectable melanoma has demonstrated significant prognostic value. Preoperative ctDNA detection has been associated with greater tumour burden and biological aggressiveness [85,86]. Postoperatively, ctDNA testing—including after completion of adjuvant therapy—can reveal minimal residual disease [87,88,89,90,91]. Moreover, ctDNA positivity following surgery strongly predicts shorter recurrence-free survival [88,92]. Longitudinal monitoring of ctDNA during follow-up allows detection of recurrence prior to clinical or radiological manifestation, offering a minimally invasive tool for real-time surveillance [90,93].

The concept of MRD monitoring is already well established in hematologic malignancies. Several studies in colorectal, breast, and lung cancers have demonstrated that perioperative ctDNA detection is associated with unfavourable prognosis [94,95,96].

In conclusion, ctDNA analysis offers a promising tool for the prognostic and predictive assessment of melanoma patients. Its integration into clinical practice may enhance early detection, risk stratification, and treatment monitoring, ultimately improving patient outcomes.

#### Significance

Circulating tumour DNA (ctDNA) offers a minimally invasive tool to assess residual disease, anticipate relapse, and refine risk stratification in melanoma. Our systematic review demonstrates that perioperative ctDNA detection consistently correlates with advanced stage, recurrence risk, and inferior survival, often predicting relapse months before clinical or radiological evidence. These findings underscore ctDNA’s potential to complement current staging and surveillance approaches, bridging basic insights into tumour biology with clinical decision-making. Establishing ctDNA as a standardized perioperative biomarker could transform melanoma management by enabling earlier intervention and more personalized follow-up strategies.

#### Research Highlights

Perioperative ctDNA detection predicts melanoma recurrence and survival.

ctDNA clearance after surgery correlates with durable remission.

Standardized trials are needed to validate ctDNA for clinical use.

## 2. Materials and Methods

**Figure 1:**
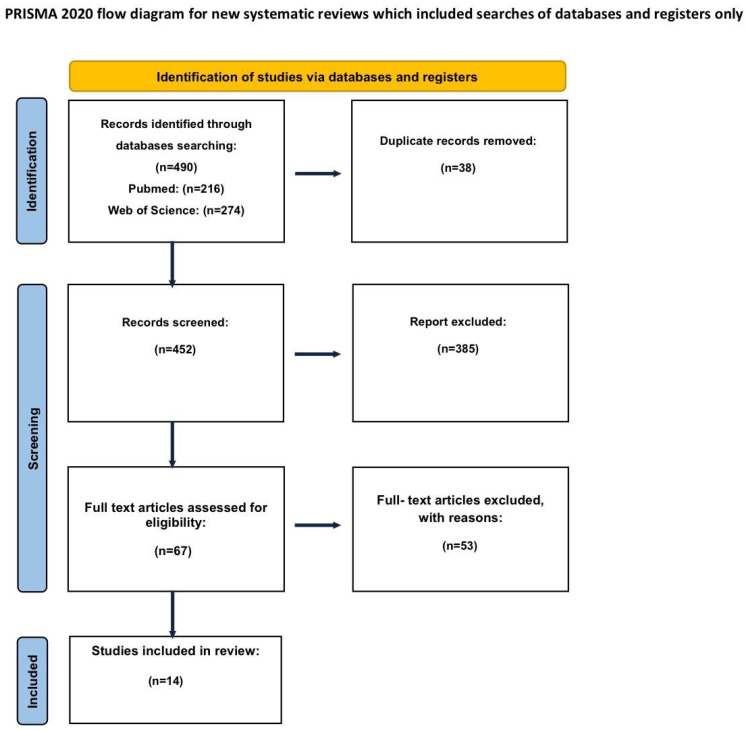
PRISMA flowchart.

This study was conducted in accordance with the Preferred Reporting Items for Systematic Reviews and Meta-Analyses (PRISMA) 2020 guidelines (Supplementary Materials) (Figure 1). The protocol was prospectively registered in the PROSPERO database (registration ID: CRD420251115512).

A systematic search of PubMed and Web of Science databases was performed for studies published between January 2017 and February 2025. The following search query was used: (“melanoma”) AND (“circulating tumour DNA” OR ctDNA OR “cell-free DNA” OR cfDNA). During the initial screening of titles and citations, abstracts, case reports, conference reports, letters, editorials, and articles written in languages other than English were excluded. Duplicates across databases were removed using Mendeley software version 2.141.2.

Studies were considered eligible if they evaluated circulating tumour DNA (ctDNA) as a means of assessing the completeness of primary melanoma resection or as a tool for determining melanoma stage. Studies were excluded if they did not involve perioperative ctDNA assessment (preoperative, intraoperative, or postoperative) or if their primary focus was limited to the use of ctDNA as a biomarker of systemic therapy efficacy, including immunotherapy or targeted therapy.

All identified records underwent title and abstract screening. Articles that met the inclusion criteria were retrieved for full-text review. Data extraction was carried out independently by six researchers under the supervision of the first author. Any disagreements regarding eligibility were resolved by consensus. When studies reported mixed cohorts, only the subgroup of surgically resected patients was included in the analysis.

From each eligible study, the following data was collected:

- General study characteristics (title, authors, year of publication).
- Patient characteristics (sample size, melanoma stage).
- Details on ctDNA assessment (methodology, timing of collection).
- Main findings (prognostic and predictive value of ctDNA in melanoma).

## 3. Results

Search and selection. The search identified 490 records (PubMed *n* = 216, Web of Science *n* = 274). After de-duplication and exclusion for inaccessible or non-eligible articles, 452 studies remained for abstract review. Sixty-seven full-text articles were assessed; 14 met all inclusion criteria and were included, encompassing 1077 patients. The data is presented in the PRISMA flowchart (Figure 1).

Overall findings.

In most included studies, persistence or detectability of ctDNA—either preoperatively or postoperatively—was strongly associated with poorer clinical outcomes [23,88,95,97,98,99,100,101,102,103,104,105,106,107,108] (Table 1).

- Preoperative prognosis. Preoperative ctDNA positivity aligned with higher stage, greater nodal involvement (including extracapsular extension), and worse survival. Patients with detectable ctDNA pre-surgery had an approximately two-fold higher mortality risk than those without detectable ctDNA [88,95,97,98,99,100,101,102,103,104,105,106,107,108].
- Relapse prediction. Postoperative ctDNA detection was linked to increased relapse risk. In one study, ctDNA anticipated recurrence by a median of 128 days, while another showed ctDNA elevation up to six months prior to diagnosis. In one cohort, 30% had detectable ctDNA during follow-up and all later relapsed; only a single relapsing patient lacked concurrent ctDNA elevation. In most reports, ctDNA cleared within ~4 weeks after radical resection in patients who remained recurrence-free [88,95,97,98,99,100,101,102,103,104,105,106,107,108].
- Metastatic progression. Rising ctDNA often preceded radiologic evidence of progression, supporting its role as an early molecular signal of metastasis [90,97,99,100,101,102,103,104,105,106,107,108,109].
- Brain metastases. Sensitivity for detecting intracranial disease was lower, likely due to the blood–brain barrier limiting ctDNA shedding into the peripheral circulation [97].
- Tumour burden. ctDNA levels correlated with total tumour volume, with higher detection when lesion size exceeded ~50 mm [101,106].
- Survival. Detectable ctDNA consistently predicted inferior survival; in one study, five-year overall survival was 33% with elevated ctDNA versus 65% when ctDNA was undetectable [99,103,107,108].

## 4. Discussion

This systematic review synthesizes evidence on the prognostic and predictive value of circulating tumour DNA (ctDNA) in patients with surgically resectable melanoma. Across the 14 included studies, (1077 patients) a consistent finding was that detectable ctDNA, whether measured preoperatively or postoperatively, strongly correlated with adverse clinical outcomes, including more advanced stage at diagnosis, increased risk of relapse, and inferior survival [75,76,77,80,82,85,86,87,88,89,90,91,92,93]. Several studies demonstrated that ctDNA positivity prior to surgery was associated with higher tumour stage, nodal involvement, extracapsular extension, and worse prognosis [85,86,93]. Patients with preoperatively detectable ctDNA had up to a two-fold increased risk of death compared to ctDNA-negative patients [88,93], which is consistent with previous evidence that ctDNA reflects tumour burden and disease aggressiveness in other solid malignancies [94,95,96]. Advanced melanomas can actively influence both local and systemic homeostasis, hijacking neuroendocrine regulatory circuits to promote tumour progression and immune evasion [110]. Tumour-derived factors, including cytokines, hormones, and melanogenic intermediates, can disrupt systemic metabolic and stress responses, potentially affecting the tumour microenvironment and distant organs. These systemic alterations may not only facilitate metastasis but also modulate the release and detectability of circulating tumour DNA, linking tumour biology to minimally invasive biomarkers. Considering these mechanisms in the context of perioperative ctDNA assessment could enhance understanding of inter-patient variability and help refine risk stratification strategies. Integrating the regulation of systemic homeostasis with cellular and metabolic changes induced by melanogenesis provides a more comprehensive view of melanoma progression and its clinical implications [110].

The persistence of ctDNA following surgery was one of the strongest predictors of early recurrence; in one study, ctDNA anticipated relapse by a median of 128 days, while another reported ctDNA elevation up to six months before radiological confirmation [88,90,93]. In a cohort where 30% of patients had detectable ctDNA during follow-up, all subsequently relapsed, whereas ctDNA negativity was strongly associated with durable remission [89,91]. Importantly, most recurrence-free patients cleared ctDNA within approximately four weeks post-resection, supporting its role as a real-time biomarker of minimal residual disease [87,88]. Rising ctDNA levels frequently preceded radiologic evidence of metastatic spread [82,90], and correlations between ctDNA concentration and tumour volume were described, with higher detection rates when lesion burden exceeded 50 mm [90,93]. Melanoma demonstrates a high incidence of intracranial metastases, occurring in approximately 20–28% of patients, with a substantial proportion presenting at the time of initial diagnosis [109]. Nevertheless, ctDNA showed lower sensitivity for detecting brain metastases [82], which is likely related to the blood–brain barrier limiting ctDNA release into the peripheral circulation [90], suggesting that ctDNA should be combined with imaging for patients at risk of intracranial disease. Across multiple studies, detectable ctDNA consistently predicted inferior survival, with one large analysis reporting five-year overall survival of only 33% in ctDNA-positive patients compared with 65% in those without detectable ctDNA [92], confirming its strong prognostic relevance. The prognostic role of perioperative ctDNA has already been demonstrated in colorectal, breast, and lung cancers, where minimal residual disease detection strongly predicts relapse [94,95,96], and the evidence summarized here supports extending this paradigm to melanoma. These findings highlight that ctDNA could be a promising biomarker for perioperative risk stratification in melanoma, where preoperative positivity may identify patients at risk for early progression and postoperative monitoring may enable timely detection of minimal residual disease and relapse, potentially refining surveillance strategies and allowing earlier therapeutic interventions in high-risk patients. It is important to highlight that ctDNA sensitivity for intracranial disease remains limited, restricting its clinical scope in certain scenarios. Future prospective and standardized studies are needed to validate if ctDNA can be used as a clinical decision-making tool in melanoma. Considering, among other factors, the presence of intracranial metastases, it will likely be necessary to combine ctDNA analysis with imaging, immunological biomarkers, and molecular profiling to improve predictive accuracy, while cost-effectiveness analyses will be essential for its implementation. 

## 5. Conclusions

In conclusion, this review demonstrates that ctDNA could be a promising biomarker for monitoring resectable melanoma, with detectable ctDNA before or after surgery consistently predicting higher recurrence risk and worse survival. However, based on the current evidence, routine implementation of perioperative ctDNA assessment in clinical practice is not yet supported. 

Nevertheless, the role of ctDNA in guiding perioperative management and surveillance in melanoma should be further investigated in large, prospective clinical trials to clarify its clinical utility in both perioperative decision-making and long-term patient monitoring.

### Limitations

Nevertheless, this review has several limitations. There was substantial heterogeneity among the included studies regarding methodology, ctDNA detection methods, the timing of perioperative sampling, patient disease stages, reported endpoints and relatively small cohorts This variability limited direct comparison between studies and reduced the overall strength of the conclusions. Additionally, differences in study design and outcome reporting further constrained the ability to draw definitive conclusions regarding the clinical applicability of ctDNA analysis. Formal risk-of-bias was not performed. 

## Figures and Tables

**Table 1:** Persistence or detectability of ctDNA.

Study (First Author, Year)	Number of Patients	Preoperative Prognosis	Relapse	Metastasis Prediction	Survival Rate	Detection Method	Timing of ctDNA Sampling
Tan, 2019 [88]	162	Yes	Yes	Yes	No	ddPCR	Pre-/Postoperative
Brunsgaard, 2023 [95]	28	Yes	Yes	No	No	NGS	Pre-/Postoperative and during surveillance
Giunta, 2022 [97]	26 (resected subgroup)	No	Yes (low sensitivity)	Yes (brain metastasis = low detection)	No	NGS	Postoperative
Marchisio, 2024 [98]	32	No	Yes	No	No	ddPCR	Postoperative
Lee, 2018 [99]	161	No	Yes	Yes	Yes	ddPCR	Postoperative
Shapochka, 2018 [100]	18	No	Yes	Yes	No	NGS	Postoperative
Braune, 2020 [101]	62	Yes	Yes	Yes (tumour burden)	No	ddPRC	Pre-/Postoperative
Genta, 2024 [102]	66	No	Yes	Yes	No	NGS	Postoperative
Lee, 2019 (JH) [103]	174	Yes	Yes	No	Yes	ddPCR	Preoperative
Chen, 2022 [104]	48	Yes	Yes	No	No	NGS	Pre-surgery
Chan, 2024 [105]	40	Yes	Yes	No	No	ddPCR	Pre-treatment/Post-surgery
Linder, 2021 [106]	15	No	Yes	Yes (tumour burden)	No	ddPCR	Longitudinal
Geoffrois, 2023 [107]	165	Yes	Yes	Yes	Yes	ddPCR	Pre-surgery/Post-treatment/longitudinal
Gouda, 2022 [108]	80	No	No	No	Yes	ddPCR	Pre-/Post-surgery, longitudinal

## Data Availability

The data supporting the findings of this study is available in the published articles cited in the reference list.

## Supplementary Materials

The following supporting information can be downloaded at: https://www.mdpi.com/article/10.3390/medicina62030461/s1 [111].
